# Vegetation photosynthetic phenology dataset in northern terrestrial ecosystems

**DOI:** 10.1038/s41597-023-02224-w

**Published:** 2023-05-19

**Authors:** Jing Fang, Xing Li, Jingfeng Xiao, Xiaodong Yan, Bolun Li, Feng Liu

**Affiliations:** 1 CAS Key Laboratory of Aquatic Botany and Watershed Ecology, Wuhan Botanical Garden, Chinese Academy of Sciences, Wuhan, 430074 China; 2 Center of Plant Ecology, Core Botanical Gardens, Chinese Academy of Sciences, Wuhan, 430074 China; 3 Research Institute of Agriculture and Life Sciences, Seoul National University, Seoul, South Korea; 4 Earth Systems Research Center, Institute for the Study of Earth, Oceans, and Space, University of New Hampshire, Durham, NH USA; 5 State Key Laboratory of Earth Surface Processes and Resource Ecology, Faculty of Geographical Science, Beijing Normal University, Beijing, 100875 China

**Keywords:** Carbon cycle, Biogeography, Phenology

## Abstract

Vegetation phenology can profoundly modulate the climate-biosphere interactions and thus plays a crucial role in regulating the terrestrial carbon cycle and the climate. However, most previous phenology studies rely on traditional vegetation indices, which are inadequate to characterize the seasonal activity of photosynthesis. Here, we generated an annual vegetation photosynthetic phenology dataset with a spatial resolution of 0.05 degrees from 2001 to 2020, using the latest gross primary productivity product based on solar-induced chlorophyll fluorescence (GOSIF-GPP). We combined smoothing splines with multiple change-point detection to retrieve the phenology metrics: start of the growing season (SOS), end of the growing season (EOS), and length of growing season (LOS) for terrestrial ecosystems above 30° N latitude (Northern Biomes). Our phenology product can be used to validate and develop phenology or carbon cycle models and monitor the climate change impacts on terrestrial ecosystems.

## Background & Summary

Vegetation phenology, the cycle sequence of the vital activities, is a highly sensitive indicator of the climate impacts on terrestrial ecosystems^1–4^. Most phenology studies focus on the structural changes of plants, such as using the growth process of leaves represented by the greenness indicators^5,6^. However, these indices work well for capturing the variations in chlorophyll content or structural changes but are less sensitive to physiological changes in vegetation photosynthesis, especially for evergreen vegetation^7,8^. Besides, the vegetation indices such as the normalized difference vegetation index (NDVI) and the enhanced vegetation index (EVI) have been widely used to estimate GPP^9–11^. Recent studies found that the methods based on vegetation indices cannot capture the photosynthesis changes in some vegetation types (e.g., evergreen forests) since the greenness and photosynthesis are sometimes decoupled^12,13^. The inaccurate estimation of vegetation greenness-based phenology can lead to substantial uncertainties in estimating plant productivity and carbon sequestration^3,11,14^.

The plant photosynthetic cycle on the seasonal time scale is termed as ‘vegetation photosynthetic phenology’. Unlike the structure change by the traditional phenology, such as bud break and leaf coloring, vegetation photosynthetic phenology represents the functional aspects of plant activities^15^. Plants regulate the carbon cycle process through photosynthesis, and the changes in photosynthetic phenology have a feedback effect on climate^15^. Therefore, tracking photosynthetic phenology on large scales may provide essential clues about the carbon cycle and help understand the drivers of carbon dynamics^16^. The photosynthetic phenology definition is based on the photosynthesis transition dates extracted from the gross primary productivity (GPP) time series. Thus, the accuracy of extracted phenology metrics largely depends on the data source and resolution of GPP. For example, the coarse spatial resolution of satellite data may include the mixed cells where the mixture of species in different phenological states appear simultaneously^2^. Currently, the GPP can either be derived from Eddy Covariance (EC) flux towers at the ecosystem scale or from satellite remote sensing or modeling at the regional or global scale^17^. The EC technique, which is considered as the most accurate observation method^18^, has provided long-term GPP estimates for more than 20 years. However, the spatial distribution limits these observations, and some key areas are still underrepresented^17^. For example, recent studies report that the warming-related greening trend in Arctic and boreal regions is identified as one of the clearest examples of climate change impacts on carbon cycles in terrestrial ecosystems^19^. Arctic amplification enhances the effects of climate change on vegetation in the regions, but only a few EC sites provide public datasets in these regions^20,21^. GPP derived from satellite remote sensing can investigate large–scale phenology across the globe^22^. Recently, the emergence of satellite-based solar-induced chlorophyll fluorescence (SIF) has offered unprecedented opportunities for developing more accurate photosynthetic phenology data products on large scales^7,23–25^. SIF, a signal emitted by plant chlorophyll molecules after absorbing photosynthetically active radiation (APAR), is considered an effective tool for diagnosing terrestrial photosynthesis and a better proxy of GPP than traditional vegetation indices^8,12,26–30^. Based on the SIF product, recent studies used the relationship between the GPP and SIF to estimate the regional or global GPP (SIF-GPP)^31,32^. Previous studies reported that SIF-GPP could better capture the GPP dynamics in evergreen vegetation and dryland ecosystems than traditional vegetation indices^12,33^.

In addition, the retrieval of phenology in previous studies mainly used a logistic regression model to fit the time series of smoothed vegetation indices or GPP. The predetermined thresholds or inflection points (e.g., using the peaks in the second derivative as the points) are identified as the transition dates of phenology in the fitted curve^6,34,35^. However, this method needs to reconstruct the original data sequence using a double-sigmoidal logistic model and thus results in uncertainty from the model parameterization^36^. Furthermore, this method usually captures a single growing season instead of multiple growing seasons in a given year^6^. Correspondingly, Richardson, *et al*.^37^ proposed a method that combined smoothing spline and multiple change-point detection to retrieve the phenology transition dates from the camera data. They constructed the PhenoCam network to automate the monitoring of canopy phenology in North America using near-surface remote sensing (i.e., cameras). Their method has excellent strength in two aspects: (1) it is not limited by the uncertainty of additional model parameters; (2) it can also be applied in ecosystems having multiple growing seasons. The method has been successfully used at multiple sites in North America^37^ and can be further extended to large scales.

Here, we aim to generate photosynthetic phenology metrics dataset based on the GPP product derived from satellite SIF data. Our data can detect multiple growing seasons, which can be used to evaluate the photosynthesis activity of vegetation on large scales. The metrics include the start state-transition dates of photosynthesis (SOS), the end state-transition dates of photosynthesis (EOS), and the duration length of photosynthesis (LOS). With this goal, we developed a method combining a smoothing filter and change-point detection to retrieve photosynthetic phenology from a recently developed SIF-based GPP product (GOSIF-GPP: 2001–2020) with a 0.05° spatial resolution. This method enables us to acquire multiple photosynthesis activity periods of vegetation within one year. The remainder of this paper describes the GOSIF-GPP and land cover data, the adopted method for retrieving photosynthetic phenology metrics, the results and discussion of the metrics and their uncertainties, and the conclusions.

## Methods

### SIF-GPP Data

**Fig. 1:**
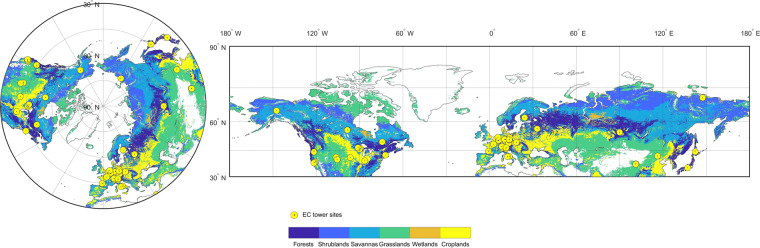
The spatial distribution of vegetation types and EC tower sites under the different projections in the Northern Biomes (0.05o spatial resolution). The left part uses the projection of Equidistant Azimuthal, and the right part uses the projection of Bolshoi Sovietskii Atlas Mira.

We used the GOSIF-GPP dataset from 2001–2020 to derive the large-scale phenology metrics in this study (http://data.globalecology.unh.edu/)^31^. GOSIF-GPP was estimated from the GOSIF dataset based on eight linear SIF-GPP relationships with 0.05° spatial and 8-day temporal resolutions (i.e., 46 GPP estimates per year for each 0.05° grid cell). The GOSIF dataset used discrete SIF soundings from the Orbiting Carbon Observatory-2 (OCO-2), remote sensing data from MODIS, and reanalysis data from MERRA-2 based on the machine learning method^38^. The GOSIF-GPP showed good seasonal and spatial patterns and was highly correlated with GPP from FLUXNET^31^. Here, we identified the vegetation type of each grid cell according to the International Geosphere-Biosphere Programme (IGBP) classification from the MODIS Land Cover Type Product Version 6 (Fig. 1, 0.05° spatial resolution). The current study used six broad vegetation types (i.e., **forests**: evergreen needleleaf forests, evergreen broadleaf forests, deciduous needleleaf forests, deciduous broadleaf forests, and mixed forests; **shrublands**: closed canopy shrublands and open shrublands; **savannas**: savannas and woody savannas; **grasslands**; **wetlands**; **croplands**) in the Northern Biomes. To reduce noise generated by non-vegetation signals, we excluded the area covered with bare soil and sparse vegetation (i.e., 8-day maximum GPP over 2001–2020 was lower than 2.0 g C m^−2^ day^−1^)^39^. Since the seasonal variation of vegetation photosynthesis in the tropical region is relatively small^2^, we focused on the area above 30° N latitude. The final dataset provided the 0.05° grid for 20 years in the six terrestrial ecosystems of the Northern Hemisphere (>30° N).

To evaluate phenology estimates based on GOSIF-GPP, we used the daily GPP data from EC flux towers across the Northern Biomes based on the Variable Ustar Threshold (VUT) mean values from the FLUXNET2015 dataset (https://fluxnet.org/data/fluxnet2015-dataset/)^40^. The FLUXNET2015 dataset provided GPP observations based on both nighttime and daytime approaches. We used the GPP average from the two methods. We retained the EC flux sites that were relatively homogeneous because the footprint of 0.05° GOSIF product and EC tower may not exactly match^31^. In this study, one given site was considered homogeneous when the dominant land cover type in 1 × 1 0.05° pixel was similar to that of the site. We selected the flux sites having available GPP data for more than one year. The selected flux tower GPP dataset includes 49 sites with 389 site-year data (detailed information on these flux sites can be found in Table S1 and Fig. 1). Here, we chose GOSIF-GPP for consistent comparison with other GPP products because the GOSIF and GOSIF-GPP data were linearly correlated in each pixel and the extracted phenological metrics based on GOSIF-GPP were the same as those based on GOSIF. We also compared the performance of GOSIF-GPP-based phenology metrics with those based on the vegetation indices and GPP products from the MODIS datasets. For each site, we extracted and calculated three vegetation indices from the Nadir Bidirectional Reflectance Distribution Function (BRDF)-Adjusted Reflectance dataset MCD43A4 (produced 1-day temporal and 500 m spatial resolution), including the NDVI, the EVI, and the near-infrared reflectance of vegetation (NIR_V_)^41^; the 8-day, 500 m MODIS-GPP data^42^; the 8-day, 0.05° GLASS-GPP data^43^; and the 8-day, 1 km BESS-GPP data^44^. The MODIS-GPP data was extracted from the MOD17A2H dataset, and it was generated by Moderate Resolution Imaging Spectroradiometer (MODIS) Leaf Area Index (LAI)/Fraction of Photosynthetically Active Radiation (FPAR). The GLASS-GPP data was generated by a light use efficiency (EC-LUE) model and the environmental variables (i.e., atmospheric CO_2_ concentration, radiation components, and atmospheric vapor pressure deficit)^43^. The BESS-GPP data was generated by a simplified process-based model, the Breathing Earth System Simulator (BESS), and MODIS Atmosphere and Land products^44^. The period of all data was consistent with the observations of EC towers.

### Photosynthetic phenology metrics

The phenology metrics in this study include SOS, EOS, and LOS. Unlike the traditional phenological events from the structural changes of leaf or flower, photosynthetic phenology is defined as the start (i.e., SOS) and end (i.e., EOS) state-transition dates of the photosynthesis cycles. These transition dates are used as the phenology metrics. One full cycle generally has five distinctive stages, including (1) photosynthesis dormancy period, a season before the growing season; (2) photosynthesis development period, a GPP rising stage; (3) photosynthesis peak period, a peak stage of GPP; (4) photosynthesis recession period, a GPP falling stage; and (5) photosynthesis dormancy period, the photosynthetically inactive stage after the growing season. Most previous studies used sigmoid-based methods (e.g., the double-logistic model) to extract the phenology, but these methods are limited to a single cycle^6^. Because some regions or ecosystems had multiple cycles in one year, we used (1) the smoothing splines to minimize the influence of outliers; (2) the change points to identify the transition dates of photosynthesis. In this study, all transition dates were extracted from the daily GPP sequence of each grid cell. Thus, we interpolated the 8-day GOSIF-GPP data to the daily scale using cubic spline interpolation before the extraction. For the cubic spline interpolation, a tridiagonal linear system (possibly with several right-hand sides) was solved for the information needed to describe the coefficients of the various cubic polynomials that made up the interpolating spline (the detailed information could be seen in the ‘spline’ method in Matlab).

**Fig. 2:**
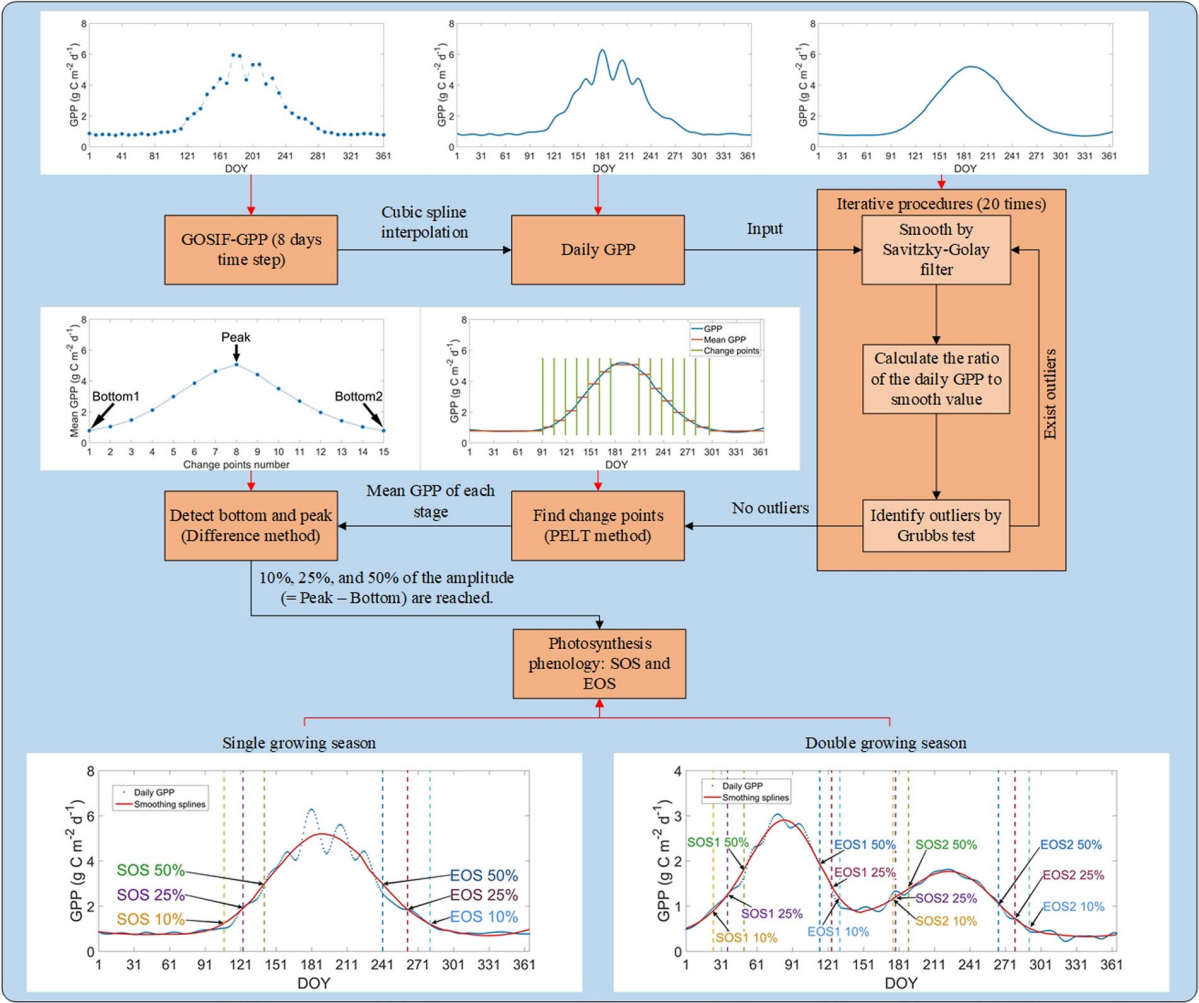
Illustration of the method for identifying the transition dates of photosynthetic phenology. The method is based on three thresholds, 10%, 25%, and 50%. Bottom1: a baseline for dormancy season before the growing season; Peak: the peak value in one single cycle; Bottom2: a baseline for dormancy season after the growing season; GPP: gross primary productivity; SOS: start time of the growing season; EOS: end time of the growing season; LOS: length of the growing season; PELT: Pruned Exact Linear Time. The example of the single growing season is from one forest site (latitude: 60.0° N, longitude: 15.5° E); the example of the double growing season is from one cropland site (latitude: 36.5° N, longitude: 36.0° E).

We constructed an automatic method to retrieve transition dates (i.e., SOS and EOS) of photosynthetic phenology using GPP data. The algorithm of this method is outlined in the flowchart in Fig. 2. The critical basis for acquiring phenological events was the data reconstruction using smoothing methods to minimize the impact of abnormal values^45^. We applied the iterative procedure to conduct the smoothing process (Fig. 2): (1) Smoothing the GPP time series by the Savitzky-Golay filter, which can reduce the influence of outliers and retain the major change characteristics of the original data sequence^46^; (2) Calculating the ratio of the daily GPP value to the smooth value; (3) Identifying outliers in these ratios by using the Grubbs test and using the smooth value instead of the daily GPP value when the ratios were larger than one standard deviation below or above the mean ratio; (4) Applying the iterative procedure up to 20 times or until no outliers were detected from one iteration to the next. This procedure can largely keep the raw seasonal pattern of photosynthesis.

The potential change points in the final smoothing splines were identified with the Pruned Exact Linear Time (PELT) method. This method can accurately detect the significant change points in the data time series and does not need to preset the number of change points^37^. For each photosynthesis cycle, we followed Richardson, *et al*.^37^ to set the penalty factor and the minimum segment length of PELT as 0.5 and 14 days, respectively. The penalty factor was used to limit the number of returned significant changes by applying the additional penalty to each prospective changepoint. The minimum segment length regulated the minimum number of days between the changepoints. Killick, *et al*.^47^ first used the PELT method, describing the calculation processes and how to find the change points in time series in detail. We calculated the mean GPP value of the adjacent change points as the potential peak and bottom baseline in one full cycle. According to the time series of mean GPP value, we used the difference method to detect the bottoms and peaks (i.e., the minimum and maximum value in each cycle). The adjacent bottoms and one peak were formed as a full cycle, and the value of these points was considered as the baselines. The difference between the baselines was used as the amplitude. Some GOSIF-GPP data affected by the weak vegetation SIF signals could have unreliable cycles. These cycles with peaks less than 0.25 of the maximum peak were excluded from the current study.

Here, the SOS and EOS dates of each cycle were determined by amplitude thresholds (i.e., the value reached 10%, 25%, and 50% of the amplitude). The amplitude was equal to the peak baseline minus the bottom baseline. Although the “true” onset of photosynthesis may correspond most closely to the 10% amplitude threshold^11^, the most tightly-constrained transition dates (i.e., the accurate transition dates) tended to occur in the later dates of the GPP rising stage and the earlier dates of the GPP falling stage^37^. Thus, we followed Richardson, *et al*.^37^ to provide the SOS and EOS dates using three amplitude thresholds: 10%, 25%, and 50%. The SOS and EOS were determined when the GPP smoothing splines reached the value of amplitude thresholds, and the LOS was defined as EOS minus SOS:

1 $$SO{S}_{i}=t,if\;GP{P}_{S}\left(t\right)=(Peak-Botto{m}_{1})\times threshol{d}_{i}$$

2 $$EO{S}_{i}=t,if\;GP{P}_{S}\left(t\right)=(Peak-Botto{m}_{2})\times threshol{d}_{i}$$

3 $$LO{S}_{i}=EO{S}_{i}-SO{S}_{i}$$

where *i* is the threshold (10%, 25%, and 50%); *t* is the day of the year (DOY); *GPP*_*S*_ is the daily value of the smoothed splines; *Bottom*_*1*_ is the baseline for dormancy season before the growing season; *Bottom*_*2*_ is the baseline for dormancy season after growing season. Note that we retrieved the phenology of vegetation indices (i.e., daily data), MODIS-GPP (i.e., interpolating the 8-day data to the daily scale), GLASS-GPP (i.e., interpolating the 8-day data to the daily scale), BESS-GPP (i.e., interpolating the 8-day data to the daily scale), and observed GPP from EC tower (i.e., EC-GPP, daily data) by using the same method.

## Data Records

**Fig. 3:**
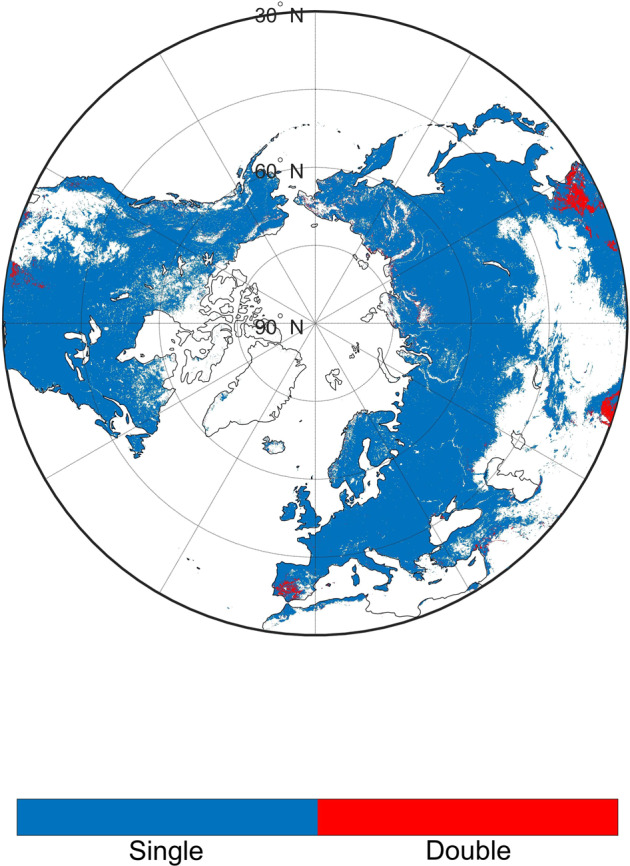
The spatial distribution of the number of growing seasons in the Northern Biomes (0.05° spatial resolution). The double seasons mean there are two photosynthesis cycles in one year. We used the Pruned Exact Linear Time (PELT) method to detect the change points of each photosynthesis cycle.

**Fig. 4:**
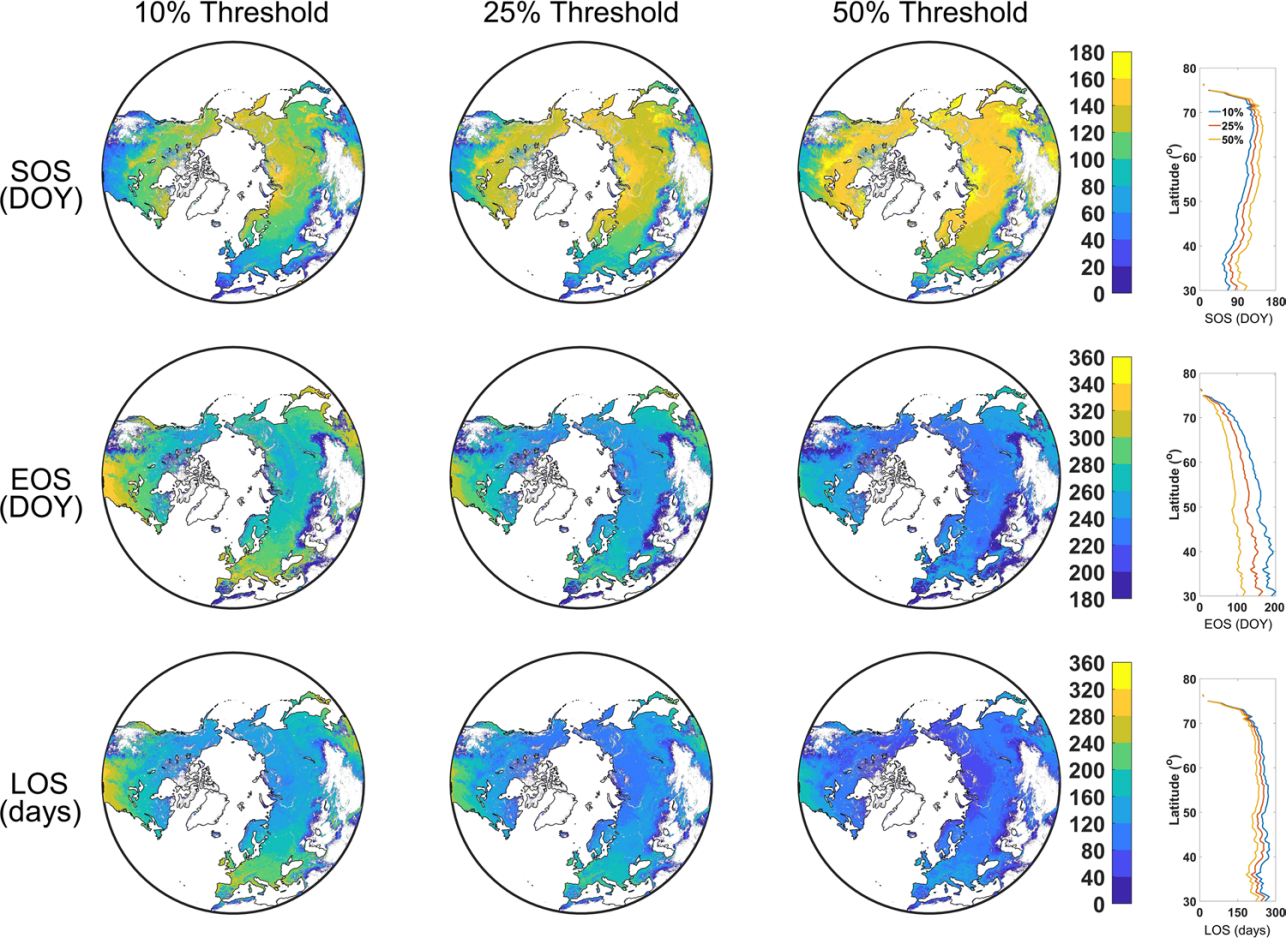
The spatial distribution of the mean photosynthetic phenology metrics (first growing season) in the Northern Biomes of 2001–2020 (0.05° spatial resolution). SOS: start time of the growing season; EOS: end time of the growing season; LOS: length of the growing season; DOY: day of the year. The right parts are the latitudinal pattern.

**Fig. 5:**
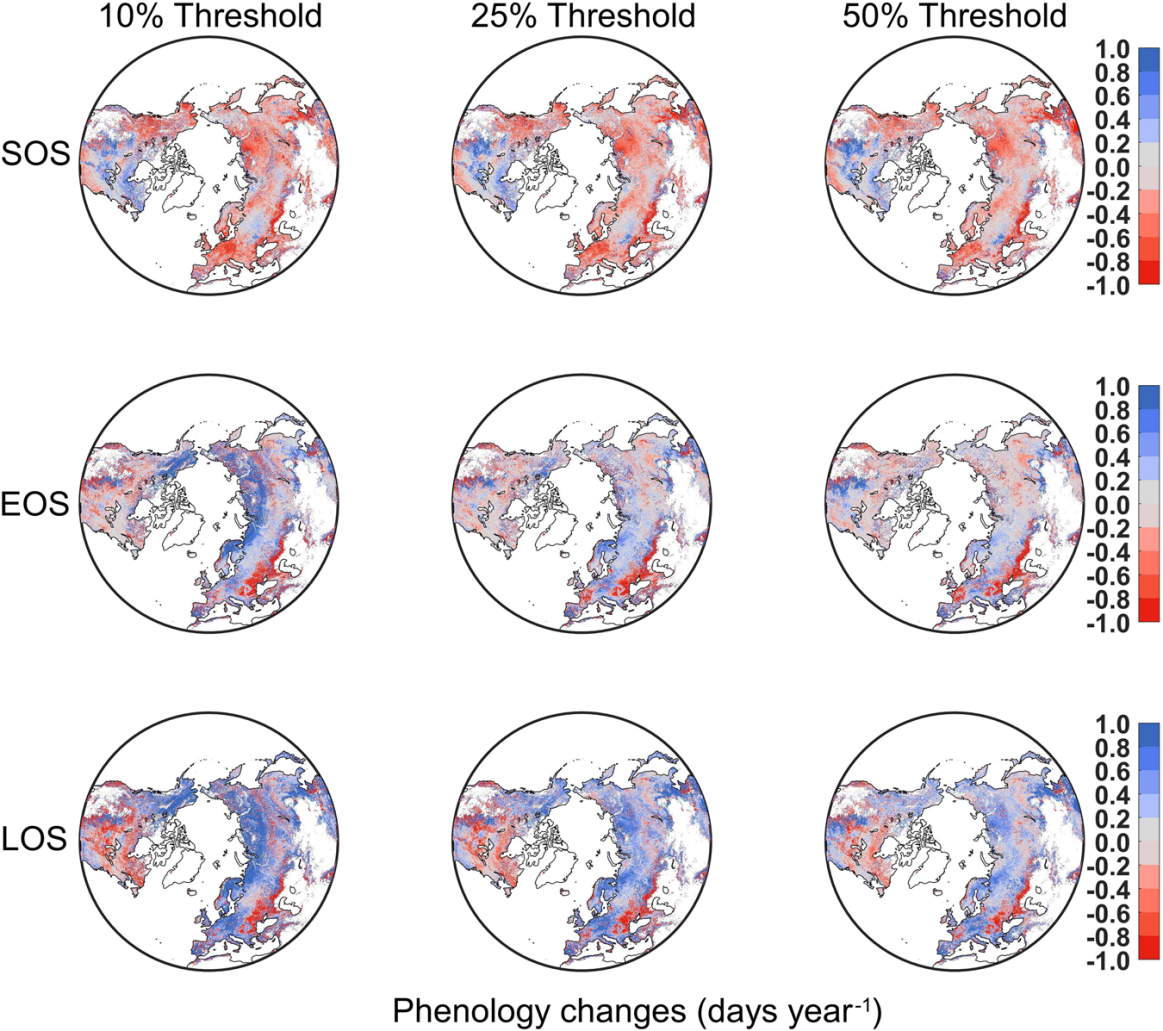
Changes in photosynthetic phenology metrics in the Northern Biomes from 2001 to 2020. SOS: start time of the growing season; EOS: end time of the growing season; LOS: length of the growing season.

During 2001–2020, our product provides annual 0.05 degrees vegetation photosynthetic phenology (i.e., start of season-SOS; end of season-EOS; length of season-LOS) in terrestrial ecosystems of the Northern Hemisphere (latitude > 30°N). The entire dataset^48^ is deposited at the open-access repository Figshare (10.6084/m9.figshare.17195009.v3). This dataset is divided into single and double growing seasons. The files include two types: the first growing season in ecosystems of the single and the double growing season; the second growing season in ecosystems of the double growing season. Figure 3 showed the spatial distribution of the number of growing seasons. Most regions in the Northern Biomes had a single growing season, while some croplands had a double growing season in a given year. We showed the spatial distribution of the first growing season in Fig. 4. For different ecosystems (Table 1), grasslands showed the earliest SOS and EOS among all biomes; forests and savannas had the latest SOS; croplands and forests exhibited the latest EOS and the longest LOS, while shrublands and grasslands had the shortest LOS. Fig. S1 showed the spatial distribution of the second growing season. Figure 5 presented the linear regression analysis using the transition dates of phenology and the time series in each grid cell, and the regression coefficient was considered as the changing trend of the grid cell.

**Table 1:** The mean value and uncertainty of photosynthetic phenology metrics in the different terrestrial ecosystems.

Terrestrial ecosystems	Threshold	Mean SOS (uncertainty)	Mean EOS (uncertainty)	Mean LOS (uncertainty)
Forests	10%	108.26 (7.34)	271.29 (18.39)	163.03 (25.73)
	25%	122.12 (8.28)	255.40 (17.31)	133.28 (25.59)
	50%	138.44 (9.38)	236.24 (16.01)	97.80 (25.40)
Shrublands	10%	75.10 (5.09)	144.33 (9.78)	69.22 (14.88)
	25%	80.97 (5.49)	137.14 (9.30)	56.17 (14.79)
	50%	88.14 (5.97)	129.02 (8.75)	40.89 (14.72)
Savannas	10%	106.37 (7.21)	244.25 (16.56)	137.88 (23.77)
	25%	117.25 (7.95)	230.10 (15.60)	112.85 (23.55)
	50%	130.85 (8.87)	213.10 (14.45)	82.24 (23.32)
Grasslands	10%	56.72 (3.84)	153.48 (10.40)	96.76 (14.25)
	25%	65.21 (4.42)	140.37 (9.52)	75.16 (13.94)
	50%	74.71 (5.06)	127.55 (8.65)	52.84 (13.71)
Wetlands	10%	106.58 (7.23)	213.13 (14.45)	106.55 (21.67)
	25%	115.24 (7.81)	202.70 (13.70)	86.83 (21.51)
	50%	126.24 (8.56)	189.61 (12.85)	63.37 (21.41)
Croplands	10%	86.60 (5.87)	272.47 (18.47)	185.87 (24.34)
	25%	102.20 (6.93)	250.78 (17.00)	148.58 (23.93)
	50%	120.56 (8.17)	226.01 (15.32)	105.45 (23.49)

Each RAR file includes the SOS, EOS, or LOS of every year (format:*.nc*, NetCDF). The file names are structured according to the file naming scheme “*<year number> <name of phenology metrics>.nc*”. In each*.nc* file, including: <*lat*>, latitudes; <*lon*>, longitudes; <*name of phenology metrics*> <*10%*>, Phenology metrics with 10% threshold; <*name of phenology metrics*> <*10%*> <*low*>, 5th percentile phenology metrics with 10% threshold; <*name of phenology metrics*> <*10%*> <*up*>, 95th percentile phenology metrics with 10% threshold; <*name of phenology metrics*> <*25%*>, Phenology metrics with 25% threshold; <*name of phenology metrics*> <*25%*> <*low*>, 5th percentile phenology metrics with 25% threshold; <*name of phenology metrics*> <*25%*> <*up*>, 95th percentile phenology metrics with 25% threshold; <*name of phenology metrics*> <*50%*>, Phenology metrics with 50% threshold; <*name of phenology metrics*> <*50%*> <*low*>, 5th percentile phenology metrics with 50% threshold; <*name of phenology metrics*> <*50%*> <*up*>, 95th percentile phenology metrics with 50% threshold.

The presented dataset is Version 3. You can extract the areas from the*.nc* files if you are only interested in a specific area (area of interest).

## Technical Validation

### Comparison with phenology derived from vegetation indices, MODIS-GPP, GLASS-GPP, BESS-GPP, and EC tower data

**Fig. 6:**
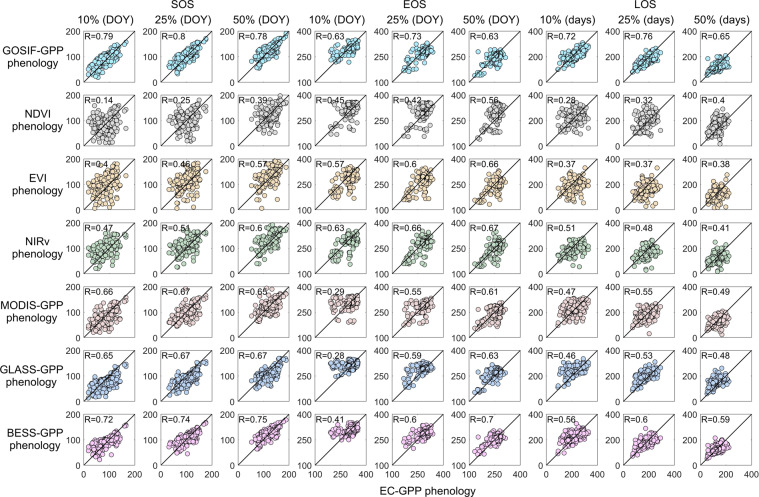
The comparison of the phenology metrics retrieves from EC tower GPP (EC-GPP) and GOSIF-GPP, NDVI, EVI, NIR_V_, MODIS-GPP, GLASS-GPP, and BESS-GPP. Each subplot has 389 site-year data. The significant correlations of all results are less than 0.05 (*p* < 0.05). The solid line represents a 1:1 line. SOS: start time of the growing season; EOS: end time of the growing season; LOS: length of the growing season; DOY: day of the year; R: correlation coefficient.

We used the photosynthetic phenology metrics extracted from the daily GPP of the flux towers to examine the corresponding metrics extracted from the GOSIF-GPP product. We also use the same method to retrieve phenology from the NDVI, EVI, NIR_V_, MODIS-GPP, GLASS-GPP, and BESS-GPP for the EC tower sites. The period of all comparison data was consistent with the EC data (these periods could be found in Table S1). According to the different thresholds, the metrics were divided into nine groups (SOS_10%_, SOS_25%_, SOS_50%_: SOS with 10%, 25%, and 50% amplitude threshold; EOS_10%_, EOS_25%_, EOS_50%_: EOS with 10%, 25%, and 50% amplitude threshold; EOS_10%_, EOS_25%_, EOS_50%_: EOS with 10%, 25%, and 50% amplitude threshold) (Fig. 6, Tables 2, 3).

**Table 2:** Correlation of the phenology metrics retrieved from EC tower GPP and GOSIF-GPP, NDVI, EVI, NIR_V_, MODIS-GPP, GLASS-GPP, and BESS-GPP.

Data source	SOS 10%	SOS 25%	SOS 50%	EOS 10%	EOS 25%	EOS 50%	LOS 10%	LOS 25%	LOS 50%
	*R*								
GOSIF-GPP	**0.79**	**0.80**	**0.78**	**0.63**	**0.73**	0.63	**0.72**	**0.76**	**0.65**
NDVI	0.14	0.25	0.39	0.45	0.42	0.56	0.28	0.32	0.40
EVI	0.40	0.46	0.57	0.57	0.60	0.66	0.37	0.37	0.38
NIRv	0.47	0.51	0.60	**0.63**	0.66	0.67	0.51	0.48	0.41
MODIS-GPP	0.66	0.67	0.65	0.29	0.55	0.61	0.47	0.55	0.49
GLASS-GPP	0.65	0.67	0.67	0.28	0.59	0.63	0.46	0.53	0.48
BESS-GPP	0.72	0.74	0.75	0.41	0.60	**0.70**	0.56	0.60	0.59

10%, 25%, and 50% mean the amplitude thresholds. The bold means the highest *R*.

**Table 3:** Root mean square error (*RMSE*) and mean bias (*Bias*) of the phenology metrics retrieved from EC tower GPP and GOSIF-GPP, NDVI, EVI, NIR_V_, MODIS-GPP, GLASS-GPP, and BESS-GPP.

Data source	SOS 10%	SOS 25%	SOS 50%	EOS 10%	EOS 25%	EOS 50%	LOS 10%	LOS 25%	LOS 50%
	*RMSE* (days)								
GOSIF-GPP	**18.03**	**15.83**	**14.99**	**23.55**	21.89	24.38	**33.93**	**29.14**	27.89
NDVI	36.91	32.18	26.20	34.13	39.68	41.86	53.87	53.92	52.39
EVI	36.97	31.34	24.97	31.75	29.41	26.09	58.53	49.04	39.28
NIRv	29.86	26.00	21.74	27.26	25.67	24.95	46.11	40.03	35.12
MODIS-GPP	22.98	20.56	18.83	30.22	24.76	23.88	43.79	36.33	32.25
GLASS-GPP	25.15	23.47	22.17	29.09	23.8	24.07	46.88	41.11	39.41
BESS-GPP	20.04	17.43	15.24	25.7	**21.59**	**19.16**	38.23	32.21	**26.61**
***Bias*** **(days)**									
GOSIF-GPP	−3.73	−**2.72**	−**1.31**	9.05	10.55	10.05	12.78	13.26	11.36
NDVI	−**0.98**	4.08	4.27	−10.94	−19.96	−26.9	−9.9	−24.0	−31.2
EVI	−17.84	−12.04	−8.33	11.89	5.27	0.77	29.74	17.31	9.10
NIRv	−13.68	−9.53	−6.98	9.17	3.67	**0.20**	22.85	13.19	7.18
MODIS-GPP	6.37	5.47	4.0	1.60	2.30	3.27	−4.77	−**3.18**	−**0.73**
GLASS-GPP	12.67	13.47	13.53	−7.29	−7.51	−9.54	−19.9	−21.0	−23.0
BESS-GPP	−4.02	−3.39	−2.43	**0.88**	**1.03**	0.94	**4.90**	4.42	3.37

10%, 25%, and 50% mean the amplitude thresholds. The bold means the lowest value.

Overall, the phenology metrics of GOSIF-GPP showed the highest correlations with the phenology metrics of EC tower GPP, while the phenology of NDVI showed the lowest correlations. For each metric, (1) SOS, the GOSIF-GPP had the best correlation coefficient (*R* = 0.78–0.80, calculated across all the site-years), the lowest root mean square error (*RMSE* = 14.99–18.03 days), and the lowest mean bias (*Bias*, from −3.73 to −1.31 days) in the 10%, 25%, and 50% thresholds. (2) EOS, the highest *R* between 10%, 25%, and 50% EOS of EC tower GPP and other data was GOSIF-GPP (0.63–0.73), and the lowest *R* was NDVI (0.42–0.56). (3) LOS, the highest *R* between 10%, 25%, and 50% EOS of EC tower GPP and other data was GOSIF-GPP (0.65–0.76), and the lowest *R* was NDVI (0.28–0.40). The comparisons indicated that GOSIF-GPP consistently performed better than the vegetation indices (i.e., NDVI, EVI, and NIRV) for different photosynthesis phenology metrics and thresholds. MODIS-GPP and GLASS-GPP had larger deviations than GOSIF-GPP, highlighting the need for improvement on light use efficiency models. The BESS-GPP performs slightly worse than the GOSIF-GPP. NIRv, the product of near-infrared reflectance and NDVI, is slightly better at capturing the phenology metrics of tower GPP than EVI and NDVI.

The derived phenology of GOSIF-GPP and EC tower GPP (i.e., EC-GPP) showed a close correspondence across the 389 site-years. The best performance of the different thresholds in SOS, EOS, and LOS was 25% (*R* = 0.80, 0.73, and 0.76; *RMSE* = 15.83, 21.89, and 29.14 days; *Bias* = −2.72, 10.55, and 13.26 days, respectively), and the threshold of 10% had relatively low performance in SOS and EOS (*R* = 0.79 and 0.63; *RMSE* = 18.03 and 23.55 days; *Bias* = −3.73 and 9.05 days, respectively) and 50% had relatively low performance in LOS (*R* = 0.65; *RMSE* = 27.89 days; *Bias* = 11.36 days). Our results showed that our method better captured the SOS than the EOS. For example, Table S3 presented that the SOS extracted from evergreen forests performed better than the EOS. Table S2 showed the performance of the GOSIF-GPP phenology in the different terrestrial ecosystems. The GOSIF-GPP phenology had a high performance for grasslands, wetlands, and croplands (*R* > 0.84) and a moderate performance for forests and shrublands (*R* = 0.77 and 0.51, respectively).

### Uncertainties of photosynthetic phenology metrics

The uncertainties in the estimates of phenology metrics mainly arise from the gridded SIF-based GPP estimates, such as using the limited explanatory variables to acquire the gridded SIF estimates (i.e., GOSIF) and the relationship between the SIF and GPP. Previously, Li and Xiao^31^ had assessed the quality of the underlying SIF and GPP data. In this study, we used the Monte Carlo Bootstrapping method^49^ to estimate the related uncertainties of the GOSIF-GPP phenology. Bootstrapping provides valuable information about uncertainties without making assumptions about the underlying data distributions^48,50^. We used bootstrapping for each year of the individual grid cell to replace the transition dates with 100 times random uniform sampling^6^. Bootstrapping was a statistical procedure that resampled a single dataset to create many simulated samples. In this study, each grid had 100 transition dates created by the bootstrapping method. The 5th and 95th percentiles of the 100 bootstrapped data were considered as the confidence interval of the mean estimated from the original transition dates.

The uncertainty used in this study was defined as the 5^th^ and 95^th^ percentiles of the 100 Monte Carlo bootstrapping samples ranging from a few days to several weeks (Table 1). The uncertainty was the lowest for SOS and the highest for LOS; EOS had intermediate uncertainty. The highest uncertainty in LOS may be because of the compounding effect of SOS and EOS^6^. Generally, metrics of grassland**s** had the lowest uncertainty: SOS uncertainty ranged from 3.8 to 5.1 days, EOS uncertainty ranged from 8.6 to 10.4 days, and LOS uncertainty ranged from 13.7 to 14.2 days; forests have the largest uncertainty, with SOS uncertainty ranging from 7.3 to 9.4 days, EOS uncertainty from 16.0 to 18.4 days, and LOS uncertainty from 25.4 to 25.7 days.

## Supplementary information

Supplementary Information

## Data Availability

The code^51^ of processes is available at Zenodo dataset (https://zenodo.org/record/7266229#.Y19tWHZBwuU). The code needs to be run in the version of Matlab after 2018.
