# Supplementary material for: Vegetation photosynthetic phenology dataset in northern terrestrial ecosystems
Source: Sci Data. 2023 May 19;10:300. doi: 10.1038/s41597-023-02224-w (PMC10198999; doi:10.1038/s41597-023-02224-w)
Supplement: Supplementary file 1 — Supplementary Information [file 41597_2023_2224_MOESM1_ESM.docx]

**Vegetation photosynthetic phenology dataset in northern terrestrial ecosystems**

***Supplementary Information***

**Appendix S1. Statistical analysis**

To evaluate the performance of each phenology against the phenology from the EC-GPP, we used correlation coefficient (*R*), root mean square error (*RMSE*), and mean bias (*Bias*):

| $R=\frac{\sum_{i=1}^{n} (X_{i}-\bar{X})\cdot(Y_{i}-\bar{Y})}{\sqrt{\sum_{i=1}^{n} {(X_{i}-\bar{X})}^{2}\cdot\sum_{i=1}^{n} {(Y_{i}-\bar{Y})}^{2}}}$ | (S1) |
| --- | --- |
| $RMSE=\sqrt{\frac{1}{n}\cdot\sum_{i=1}^{n} {(X_{i}-Y_{i})}^{2}}$ | (S2) |
| $Bias=\frac{1}{n}\sum_{i=1}^{n} {(X}_{i}-Y_{i})$ | (S3) |

Where the *X_i_* and *Y_i_* are the phenology from each dataset and the phenology from the EC-GPP, respectively; $\bar{X}$ and $\bar{Y}$ represent their mean values; *n* is the number of the total years.

**Table S1.** The flux tower sites in the Northern Biomes.

| **Site-name** | **Vegetation type** | **Latitude** | **Longitude** | **Start year** | **End year** | **Unavailable year** |
| --- | --- | --- | --- | --- | --- | --- |
| BE-Bra | MF | 51.31 | 4.52 | 2001 | 2014 | 2003 |
| BE-Vie | MF | 50.31 | 6.00 | 2001 | 2014 | - |
| CA-Man | ENF | 55.88 | -98.48 | 2001 | 2008 | 2004, 2005 |
| CA-NS2 | ENF | 55.91 | -98.52 | 2002 | 2005 | - |
| CA-NS3 | ENF | 55.91 | -98.38 | 2002 | 2004 | - |
| CA-NS4 | ENF | 55.91 | -98.38 | 2003 | 2005 | - |
| CA-NS5 | ENF | 55.86 | -98.49 | 2002 | 2005 | - |
| CA-Qfo | ENF | 49.69 | -74.34 | 2004 | 2010 | - |
| CH-Cha | GRA | 47.21 | 8.41 | 2006 | 2014 | 2008, 2011 |
| CN-Du2 | GRA | 42.05 | 116.28 | 2008 | 2008 | - |
| CN-Ha2 | WET | 37.61 | 101.33 | 2003 | 2005 | - |
| CN-Ham | GRA | 37.37 | 101.18 | 2002 | 2004 | - |
| CZ-BK1 | ENF | 49.50 | 18.54 | 2005 | 2008 | - |
| DE-Geb | CRO | 51.10 | 10.91 | 2001 | 2014 | - |
| DE-Hai | DBF | 51.08 | 10.45 | 2001 | 2012 | - |
| DE-lkb | ENF | 49.10 | 13.30 | 2009 | 2013 | - |
| DE-Obe | ENF | 50.78 | 13.72 | 2008 | 2014 | - |
| DE-RUR | GRA | 50.62 | 6.30 | 2011 | 2014 | - |
| DE-Spw | WET | 51.89 | 14.03 | 2010 | 2014 | - |
| DK-Tha | ENF | 50.96 | 13.57 | 2001 | 2014 | - |
| FI-Hyy | ENF | 61.85 | 24.30 | 2001 | 2014 | - |
| FR-Fon | DBF | 48.48 | 2.78 | 2005 | 2014 | - |
| FR-Lbr | ENF | 44.72 | -0.77 | 2001 | 2008 | 2002 |
| IT-Col | DBF | 41.85 | 13.59 | 2001 | 2014 | - |
| IT-Cpz | EBF | 41.71 | 12.38 | 2001 | 2008 | 2003, 2008 |
| IT-Lav | ENF | 45.96 | 11.28 | 2003 | 2014 | - |
| IT-Ren | ENF | 46.59 | 11.43 | 2002 | 2013 | 2004 |
| JP-MBF | DBF | 44.39 | 142.32 | 2004 | 2005 | - |
| JP-SMF | MF | 35.25 | 137.07 | 2002 | 2006 | 2002, 2004 |
| NL-Loo | ENF | 52.17 | 5.74 | 2001 | 2013 | - |
| RU-Cok | OSH | 70.83 | 147.49 | 2003 | 2013 | - |
| RU-Fyo | ENF | 56.46 | 32.92 | 2001 | 2014 | - |
| RU-Ha1 | GRA | 54.73 | 90.00 | 2003 | 2004 | - |
| US-Blo | ENF | 38.90 | -120.63 | 2001 | 2007 | 2005 |
| US-GLE | ENF | 41.36 | -106.24 | 2005 | 2014 | - |
| US-Ha1 | DBF | 42.54 | -72.17 | 2001 | 2012 | - |
| US-Me2 | ENF | 44.45 | -121.56 | 2002 | 2014 | 2003 |
| US-ME6 | ENF | 44.32 | -121.60 | 2010 | 2013 | 2012, 2013, 2014 |
| US-MMS | DBF | 39.32 | -86.41 | 2001 | 2014 | - |
| US-Ne1 | CRO | 41.17 | -96.48 | 2001 | 2012 |  |
| US-Ne2 | CRO | 41.16 | -96.47 | 2002 | 2012 | 2002 |
| US-Ne3 | CRO | 41.18 | -96.44 | 2001 | 2012 | 2001 |
| US-NR1 | ENF | 40.03 | -105.55 | 2001 | 2014 | - |
| US-Pfa | MF | 45.95 | -90.27 | 2001 | 2014 | - |
| US-Prr | ENF | 65.12 | -147.49 | 2011 | 2013 | - |
| US-Syv | MF | 46.24 | -89.35 | 2002 | 2014 | 2004 |
| US-Ton | WSA | 38.43 | -120.97 | 2002 | 2014 | - |
| US-Tw1 | WET | 38.11 | -121.65 | 2013 | 2014 | - |
| US-Wcr | DBF | 45.81 | -90.08 | 2001 | 2014 | - |
| US-Wi3 | DBF | 46.63 | -91.10 | 2002 | 2004 | 2003 |

**Table S2.** Statistical comparison of the phenology metrics retrieved from EC tower GPP and GOSIF-GPP in the different forest ecosystems. 10%, 25%, and 50% mean the amplitude thresholds. N: number of the site-years; *R*: correlation coefficient; *RMSE*: root mean square error; *Bias*: mean bias; ENF: evergreen needleleaf forest; EBF: evergreen broadleaf forest; DBF: deciduous broadleaf forest; MF: mix forest.

|  | Vegetation types | SOS  10% | SOS  25% | SOS  50% | EOS  10% | EOS  25% | EOS  50% | LOS  10% | LOS  25% | LOS  50% |
| --- | --- | --- | --- | --- | --- | --- | --- | --- | --- | --- |
| ***R*** | ENF (N=153) | 0.82 | 0.85 | 0.81 | 0.50 | 0.38 | 0.11 | 0.80 | 0.78 | 0.65 |
|  | EBF  (N=6) | 0.17 | 0.30 | 0.15 | -0.40 | -0.48 | 0.57 | -0.39 | -0.46 | -0.06 |
|  | DBF (N=80) | 0.82 | 0.85 | 0.81 | 0.46 | 0.17 | 0.09 | 0.79 | 0.74 | 0.28 |
|  | MF  (N=56) | 0.92 | 0.93 | 0.93 | 0.75 | 0.79 | 0.70 | 0.89 | 0.92 | 0.93 |
| ***RMSE***  **(days)** | ENF (N=153) | 19.32 | 16.88 | 15.98 | 26.53 | 25.02 | 24.38 | 39.90 | 36.97 | 34.12 |
|  | EBF  (N=6) | 22.42 | 24.02 | 24.12 | 27.86 | 42.10 | 24.38 | 40.06 | 46.36 | 39.03 |
|  | DBF (N=80) | 13.85 | 13.98 | 15.24 | 9.04 | 13.84 | 24.38 | 14.25 | 11.94 | 18.91 |
|  | MF  (N=56) | 17.50 | 10.94 | 6.18 | 12.52 | 10.80 | 24.38 | 26.29 | 18.15 | 11.56 |
| ***Bias***  **(days)** | ENF (N=153) | -12.10 | -11.70 | -10.39 | 19.80 | 17.93 | 10.05 | 31.90 | 29.64 | 25.18 |
|  | EBF  (N=6) | -16.80 | -18.40 | -8.40 | -10.40 | -22.20 | 10.05 | 6.40 | -3.80 | -26.80 |
|  | DBF (N=80) | 11.17 | 11.87 | 12.81 | 2.30 | 6.64 | 10.05 | -8.87 | -5.23 | 2.23 |
|  | MF  (N=56) | -11.50 | -5.95 | -0.75 | 5.52 | 4.70 | 10.05 | 17.02 | 10.66 | 5.57 |

**Table S3.** Statistical comparison of the phenology metrics retrieved from EC tower GPP and GOSIF-GPP in the different terrestrial ecosystems. 10%, 25%, and 50% mean the amplitude thresholds. N: number of the site-years; *R*: correlation coefficient; *RMSE*: root mean square error; *Bias*: mean bias.

|  | Vegetation types | SOS  10% | SOS  25% | SOS  50% | EOS  10% | EOS  25% | EOS  50% | LOS  10% | LOS  25% | LOS  50% |
| --- | --- | --- | --- | --- | --- | --- | --- | --- | --- | --- |
| ***R*** | Forests (N=295) | 0.77 | 0.77 | 0.70 | 0.44 | 0.31 | 0.09 | 0.72 | 0.69 | 0.56 |
|  | Shrublands (N=11) | 0.51 | 0.69 | 0.69 | 0.73 | 0.76 | 0.34 | 0.98 | 1.00 | 0.80 |
|  | Savannas (N=13) | 0.28 | 0.85 | 0.92 | 0.28 | 0.20 | 0.22 | 0.39 | 0.54 | 0.74 |
|  | Grasslands (N=13) | 0.95 | 0.95 | 0.95 | 0.84 | 0.81 | 0.05 | 0.93 | 0.93 | 0.64 |
|  | Wetlands (N=10) | 0.95 | 0.99 | 0.99 | 0.46 | 0.30 | 0.38 | 0.99 | 0.91 | 0.87 |
|  | Croplands (N=47) | 0.84 | 0.77 | 0.72 | 0.49 | 0.54 | 0.60 | 0.31 | 0.42 | 0.26 |
| ***RMSE***  **(days)** | Forests (N=295) | 17.84 | 15.51 | 14.84 | 21.38 | 21.29 | 24.09 | 33.02 | 29.99 | 28.23 |
|  | Shrublands (N=11) | 31.68 | 30.60 | 30.06 | 36.89 | 28.51 | 18.39 | 19.35 | 13.95 | 4.20 |
|  | Savannas (N=13) | 23.62 | 22.86 | 19.10 | 32.32 | 31.70 | 18.98 | 31.32 | 22.77 | 12.92 |
|  | Grasslands (N=13) | 14.29 | 13.07 | 11.83 | 12.34 | 11.51 | 36.01 | 22.98 | 20.31 | 39.90 |
|  | Wetlands (N=10) | 12.77 | 10.09 | 11.30 | 10.28 | 10.49 | 10.53 | 12.69 | 16.90 | 17.55 |
|  | Croplands (N=47) | 22.92 | 20.65 | 19.41 | 48.99 | 33.21 | 25.04 | 58.41 | 32.17 | 20.68 |
| ***Bias***  **(days)** | Forests (N=295) | -6.14 | -4.87 | -2.86 | 12.44 | 12.15 | 12.30 | 18.59 | 17.01 | 15.16 |
|  | Shrublands (N=11) | -15.27 | -14.27 | -8.64 | 29.09 | 20.82 | 8.82 | 4.33 | 2.67 | -0.33 |
|  | Savannas (N=13) | 21.10 | 22.30 | 18.70 | 3.50 | 21.70 | 12.80 | -17.60 | -0.60 | -5.90 |
|  | Grasslands (N=13) | -7.94 | -5.50 | -4.44 | 2.94 | 1.19 | -12.13 | 10.88 | 6.69 | -7.69 |
|  | Wetlands (N=10) | 9.13 | 7.50 | 8.63 | -1.38 | -1.00 | -0.13 | -10.50 | -8.50 | -8.75 |
|  | Croplands (N=47) | 15.71 | 12.65 | 8.88 | -31.65 | -7.24 | -0.47 | -47.35 | -19.88 | -9.35 |

**
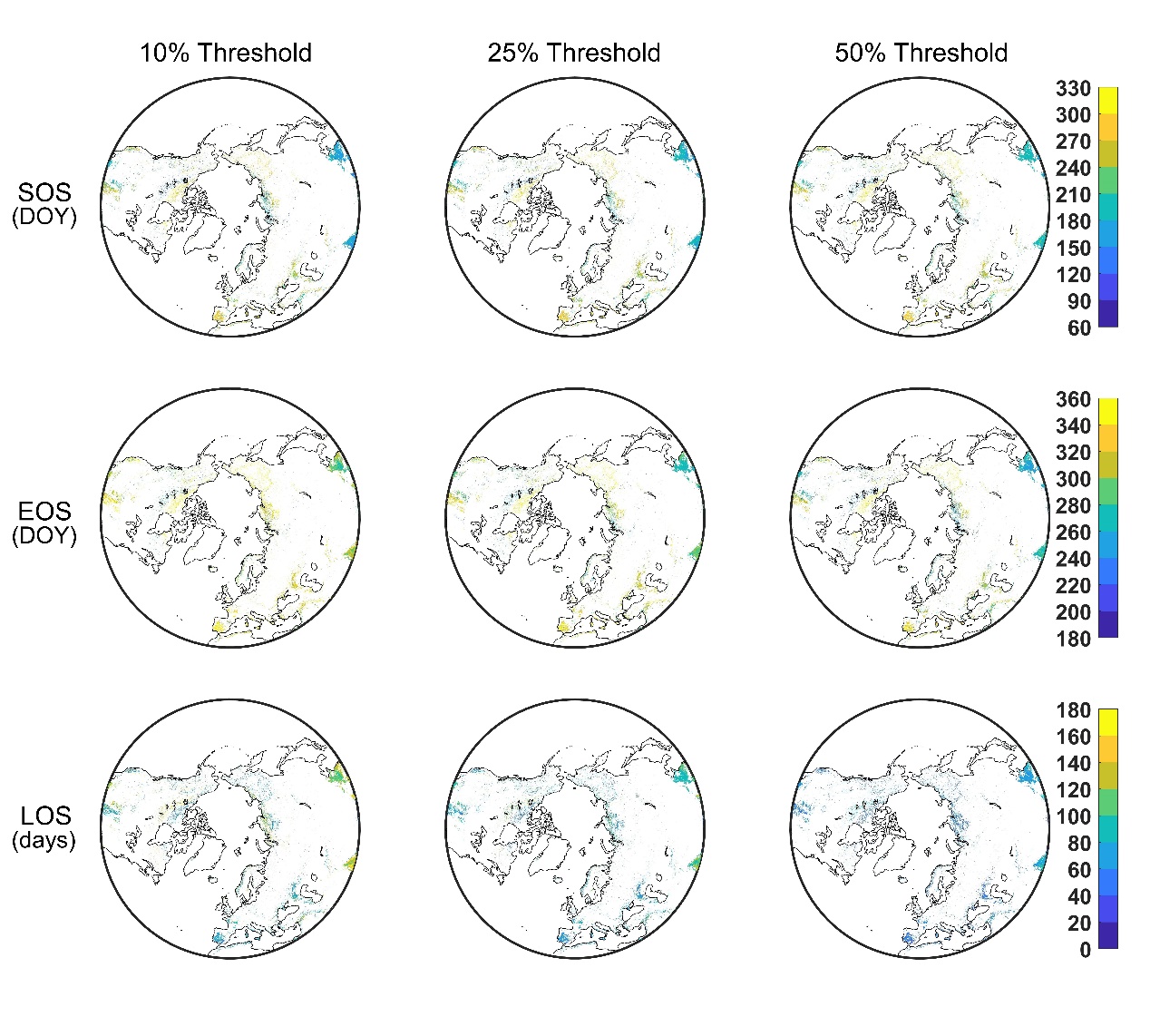
Fig. S1.** The spatial distribution of the mean photosynthetic phenology metrics of the second growing season in the Northern Biomes of 2001-2020 (0.05^o^ spatial resolution). SOS: start time of the growing season; EOS: end time of the growing season; LOS: length of growing season; DOY: day of the year.
